# Regional Cerebral Blood Flow Increase After Transcatheter Aortic Valve Replacement Is Related to Cardiac Output but Is Not Associated with Delirium: An Observational Cohort Study Using Transcranial Indocyanine Green Dye Dilution Technique

**DOI:** 10.3390/jcm14124317

**Published:** 2025-06-17

**Authors:** Maximilian Oremek, Paul Nowotny, Sebastian Zimmer, Atsushi Sugiura, Leonie Weinhold, Juerg Froehlich, Martin Soehle, André Diedrich, Marcus Thudium

**Affiliations:** 1 Department of Anesthesiology and Intensive Care Medicine, University Hospital Bonn, Venusberg Campus 1, 53127 Bonn, Germany; maximilian.oremek@ukbonn.de (M.O.); martin.soehle@ukbonn.de (M.S.); 2 Department of Cardiology and Pulmonology, University Hospital Bonn, 53127 Bonn, Germany; sebastian.zimmer@ukbonn.de (S.Z.); atsushi.sugiura@ukbonn.de (A.S.); 3 Institute of Medical Biometry, Informatics and Epidemiology, University of Bonn, 53127 Bonn, Germany; weinhold@imbie.meb.uni-bonn.de; 4 Fields and More GmbH, 8032 Zurich, Switzerland; juerg.froehlich@fieldsatwork.ch; 5 Autonomic Dysfunction Center, Division of Clinical Pharmacology, Department of Medicine, Vanderbilt University Medical Center, Nashville, TN 37232, USA; andre.diedrich@vumc.org

**Keywords:** cerebral blood flow, cerebral hyperperfusion, delirium

## Abstract

**Background:** Despite the success of transcatheter aortic valve repair (TAVR) over the past years, its impact on global and cerebral hemodynamics remains largely unexplored. Changes in cerebral blood flow may be associated with delirium, which may occur in 26 to 29% of cases. We aimed to examine the relationships between global hemodynamic parameters and cerebral parameters in patients who underwent TAVR and their impact on postinterventional delirium. **Methods:** Patients scheduled for TAVR were enrolled after obtaining written informed consent. Patients received light sedation according to standard procedures. Cerebral blood flow (CBF) was measured with a noninvasive near-infrared spectroscopy-based method using intravenous indocyanine green injection. CBF measurements were taken at the beginning of the TAVR procedure and after the valve was in place. Patients were screened for delirium using CAM-ICU and NuDESC tests before and after intervention. **Results:** A total of 52 of 60 patients remained for analysis. Thirteen patients (25%) developed delirium. Mean arterial pressure (MAP) remained unchanged, while cardiac output increased after TAVR by 44%. CBF also increased after TAVR. No significant difference was observed in CBF changes between the groups with and without delirium. A linear mixed model analysis revealed a linear relationship between CO and CBF but not between MAP and CBF. In an exploratory analysis, decreased cerebral oxygenation and increased deoxygenated hemoglobin, as measured by NIRS after TAVR, were associated with delirium. **Conclusions:** The results confirm that CO is an independent factor in CBF, while CBF changes per se are not linked to delirium. However, we found a mismatch between CBF and regional cerebral parameters, which may reflect cerebral metabolism and its relation to the development of delirium. This remains to be confirmed by further studies.

## 1. Introduction

Transcatheter cardiac valve replacement (TAVR) procedures are becoming increasingly common even in patients with lower surgical risk [1]. Despite the success over the past years, the sudden change in global and cerebral hemodynamics after TAVR remains largely unexplored. While cardiac output parameters have been reported to increase after the procedure, this may not apply to blood flow to the brain [2]. Such sudden changes to a chronically adapted system may be counterbalanced by regulatory mechanisms, such as baroreflex or cerebral autoregulation. It is possible that such hemodynamic changes are associated with postinterventional delirium.

Delirium rates after TAVR have been reported to range from 26 to 29%, compared to 33 to 51% in patients following surgical aortic valve replacement [3,4]. It has also been shown that patients with delirium after undergoing TAVR are especially prone to long-term negative outcomes [4]. The mechanism underlying the development of delirium remains unknown. We have previously proposed a connection between cerebral hyperperfusion events during cardiopulmonary bypass and postoperative delirium [5]. While cardiopulmonary bypass is not present during TAVR, valve functionality is restored within a short time, potentially resulting in hyperperfusion phenomena, especially in older patients receiving TAVR.

The purpose of this study was to examine the relationship between changes in CBF and postinterventional delirium. Cerebral blood flow (CBF) changes were measured with a novel near-infrared spectroscopy (NIRS) device using the injection of a green dye solution to calculate mean transit time, a surrogate for cerebral blood flow [6]. We further investigated factors contributing to delirium in an exploratory analysis. Finally, we aimed to examine the relationships between global hemodynamic parameters, such as blood pressure and cardiac output, and cerebral parameters, such as cerebral blood flow and cerebral saturation.

We hypothesized an increase in cerebral blood flow after valve expansion due to increased cardiac output. Furthermore, we hypothesized a connection between the increase in cerebral blood flow after TAVR and delirium, as well as a connection between pre-interventional cognitive status and initial cerebral blood flow.

## 2. Materials and Methods

This observational study was approved by the University of Bonn Ethics Committee (Chair: Prof. K. Racké, No 327/21). After providing written consent, patients scheduled for transcatheter aortic valve replacement were included. The exclusion criteria were as follows: no informed consent, intervention other than aortic valve replacement, no arterial access, and conversion to open surgery. After patient inclusion, a mini-mental state examination (MMSE) was performed to assess the initial cognitive state.

Patients received central venous catheterization the day before the procedure. On the intervention day, patients were placed on the operating table in a supine position. Monitoring consisted of electrocardiography, peripheral oxygen saturation, and EEG with bispectral index analysis (BIS). An arterial cannula was placed in the left radial artery for continuous blood pressure measurement after local anesthesia. Non-calibrated pulse-contour analysis was connected to the arterial line for advanced hemodynamic monitoring (Edwards EV1000, Edwards Lifesciences, Irvine, CA, USA). A sensor patch with a magnetically attached optic sensor for cerebral blood flow measurement by NIRS (RheoPatch, Luciole Medical AG, Zurich, Switzerland) was placed on the left forehead above the frontal lobe. The Luciole device uses an approach based on the classical dye dilution method to calculate cerebral tissue blood flow by locally measuring the intracranial mean transit time of indocyanine green (ICG) using specific algorithms to correct for extracranial contamination with resulting values of CBF and cerebral blood volume (CBV). The EV1000, the Luciole device, and basic monitoring (IACS, Draeger, Lübeck, Germany) were connected to a Moberg CNS Monitor (Moberg Research, Ambler, PA, USA) for continuous real-time data acquisition. Once conditions were stable, a baseline CBF measurement (pre-TAVR) was taken by injecting a precalculated dose of up to 25 mg of ICG via central venous access. One measurement, including washout time after injection, takes 7 min. All measurements were performed under light sedation with continuous infusion of 100–200 µg/h of remifentanil via a syringe pump and with propofol via target-controlled infusion with an effect concentration target of 1.0, aiming at a BIS value of 80. Vasopressors were not routinely started with induction but kept on standby. No further methods of neuroprotection were applied.

A transvenous pacemaker was positioned and tested by the interventionalist. After local anesthesia, access through the femoral artery was established. Retrograde positioning of the guidewire through the aortic valve was performed under X-ray imaging, once proper positioning was confirmed, the prosthetic valve was advanced over the guidewire. Prosthetic valves were either the balloon-inflated Sapien 3 (Edwards Lifesciences, Irvine, CA, USA), which required rapid pacing (heart rate of 180/min) via the external pacemaker during valve expansion, or the self-expanding Evolut Pro (Medtronic, Minneapolis, MN, USA), which required a constant heart rate of 130/min during the expansion process.

After the prosthetic valve was placed and the patient stabilized, the second ICG measurement (post-TAVR) was performed. Once good valve function was confirmed, femoral access though was removed, and the patient was transferred to either an intensive care unit or an intermediate care unit for further surveillance.

Median values of mean arterial pressure (MAP) and cardiac output (CO) were calculated during one minute before the first and second ICG measurements, respectively.

Post-interventional tests for delirium were performed on the first and third post-interventional days using the Confusion Assessment Method for Intensive Care Units (CAM-ICU) by one trained investigator. Possible delirium phases between CAM-ICU tests were screened using the Nursing Delirium Screening Scale (Nu-DESC) [7]. Delirium was considered likely if at least one of the tests indicated delirium.

### 2.1. Sample Size Estimation

We assumed an initial mean CBF value of 40 mL/kg/min and a mean CBF of 50 mL/kg/min after TAVR with a standard deviation of 16 mL/kg/min, which would require a sample size of 41 patients to reach a power of 80% with a two-sided level of significance of 5%. Values are based on previous intraoperative results using the same method [8]. While we assumed higher CBF without general anesthesia, a similar standard deviation was assumed. However, considering experiences from previous ICG measurements, we expected a large dropout rate due to failed measurements of up to 30%. Therefore, we aimed to include 60 patients.

### 2.2. Statistical Methods

The baseline characteristics of the study population are presented as the mean and standard deviation (SD) or medians and interquartile range (IQR) for continuous variables and counts, with percentages reported for categorical variables. We then analyzed the changes in cerebral and hemodynamic parameters during TAVR. The differences were investigated with respect to postinterventional delirium (yes/no) using the Wilcoxon Mann–Whitney test. The association between baseline CBF and the mini-mental status examination (MMSE) was assessed using a linear regression model. The association between CBF and MAP as well as between CBF and CO were investigated using linear mixed models, with the participant identifier as a random intercept to account for the correlation between repeated observations from the same subject (pre- and post-interventional measurements). The statistical significance level was set at a *p*-value of <0.05 (two-sided).

Additionally, we used a univariate model of cerebral, hemodynamic, and blood gas analysis parameters to discover differences between patients with and without delirium.

## 3. Results

Sixty patients with TAVR implantations were initially included. Eight patients were excluded due to missing data from failed ICG measurements or missing hemodynamic data. Of the remaining 52 patients, 13 (25%) developed delirium. All patients were categorized as ASA 3 or 4 according to the American Society of Anesthesiologists classification. The basic demographic characteristics of the patients are presented in Table 1.

In patients with delirium, cardiac output (CO) increased by 17%, compared to a 22% increase in patients without delirium. MAP decreased by 8% vs. 12% in patients with versus without delirium, respectively. CBF increased by 44% in all patients, with no significant difference between the two groups. For CBV, the difference between the pre-TAVR and post-TAVR measurements was 6% in the delirium group and 9% in the non-delirium group. The intra-group comparison showed no differences (*p* = 0.65).

**Figure 1:**
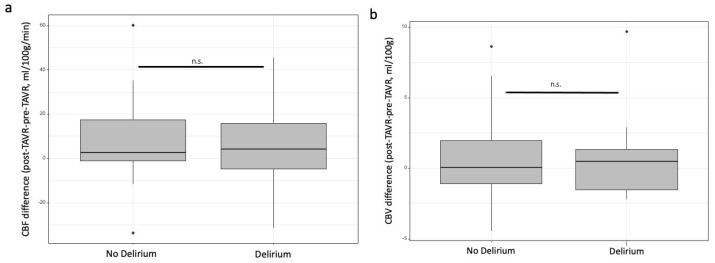
CBF (**a**). and CBV (**b**) differences before and after TAVR in the non-delirium and delirium groups. CBF: cerebral blood flow, CBV: cerebral blood volume, n.s.: non-significant.

Table 2 summarizes the patient characteristics for global and cerebral hemodynamic parameters. Figure 1 shows the box plot for cerebral parameters before and after TAVR.

**Figure 2:**
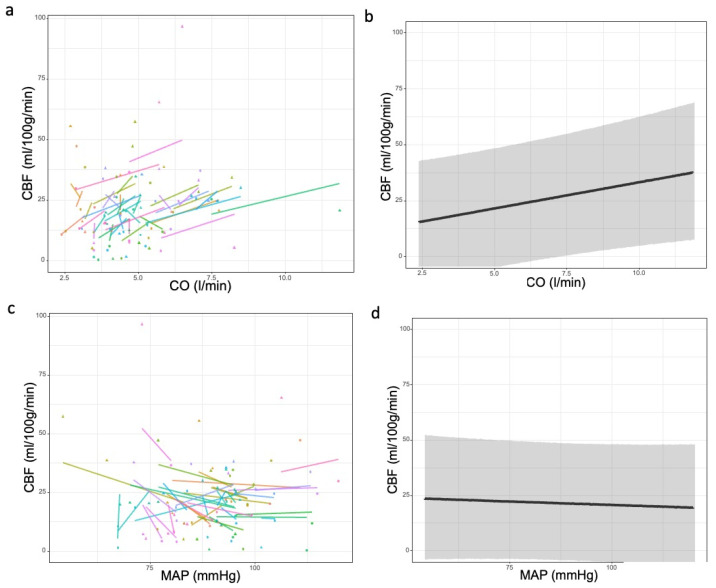
Relationship between global hemodynamic parameters (MAP, CO) and cerebral blood flow for individual patients and all patients, as calculated using a linear mixed model. (**a**,**b**): CO–CBF, (**c**,**d**): MAP–CBF, (**a**,**c**) individual model predictions comparing pre-TAVR and post-TAVR, (**b**,**d**) model prediction for the whole cohort. The results in (**b**,**d**) show a positive linear relationship between CO and CBF (estimate = 2.33, CI: 0.15–4.52, *p* = 0.037), while there was a non-significant relationship between MAP and CBF (estimate = −0.06, CI: −0.34; 0.22, *p* = 0.672), suggesting CO as an independent contributor to CBF and MAP independence of CBF. CBF: cerebral blood flow (mL/100 g/min), CO: cardiac output (L/min), MAP: mean arterial pressure (mmHg), TAVR: transcatheter aortic valve repair.

We found a relationship between pre-interventional cognitive function, as represented by the MMSE and the initial CBF measurement (estimate = 0.10, CI: 0.02–0.18, *p* = 0.015), as shown in Supplement S1. However, the correlation between MMSE and CBF was weak. A positive correlation was also found between CO and CBF (estimate = 2.33, CI: 0.15–4.52, *p* = 0.037), while a slightly negative but non-significant relationship was noted between MAP and CBF (estimate = −0.06, CI: −0.34; 0.22, *p* = 0.672) as illustrated in Figure 2.

**Figure 3:**
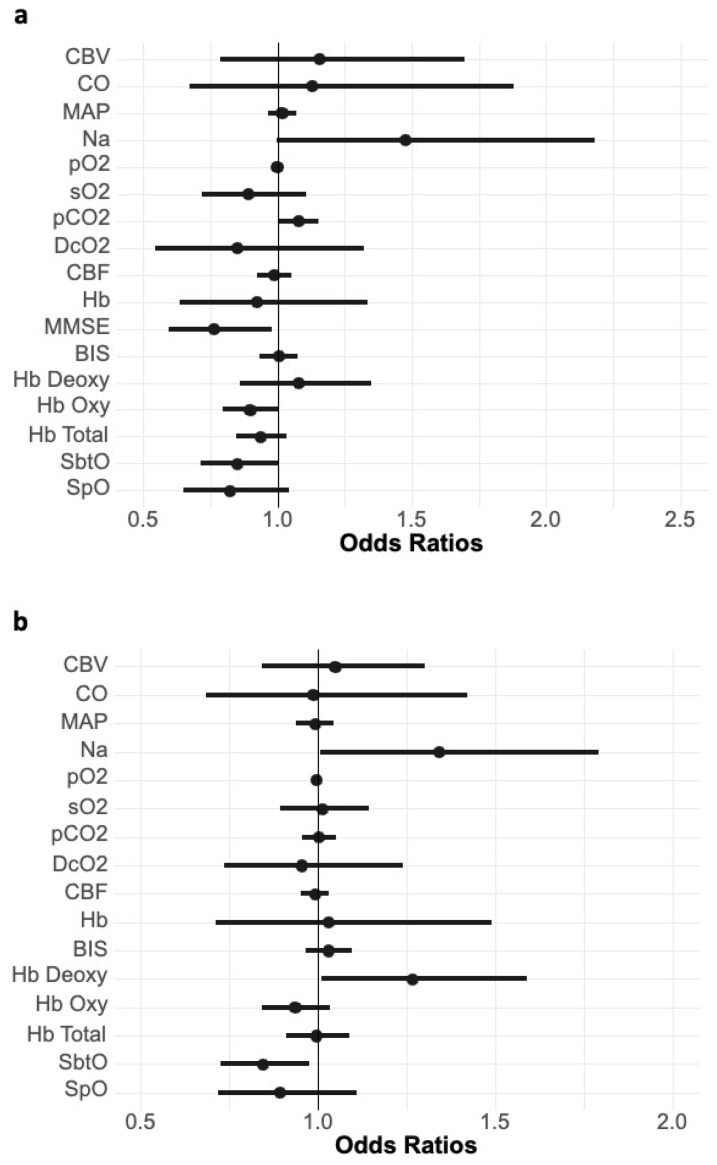
Odds ratios in relationship to postinterventional delirium (**a**) before TAVR and (**b**) after TAVR. BIS: bispectral index, CBF: cerebral blood flow, CBV: cerebral blood volume, CO: cardiac output, DcO2: cerebral oxygen delivery calculated from CBF, Hb Deoxy: deoxygenated tissue hemoglobin measured by NIRS, Hb Oxy: oxygenated tissue hemoglobin measured by NIRS, Hb Total: total tissue hemoglobin measured by NIRS, Na: continuous noradrenalin, MMSE: mini-mental state examination, pCO2: partial pressure of carbon dioxide measured by blood gas analysis, SbtO: tissue oxygen saturation measured by NIRS, sO2: blood oxygen saturation measured by blood gas analysis, SpO: peripheral oxygen saturation.

In a univariate model of measured parameters versus delirium, MMSE (*p* = 0.032) and initial pCO2 (*p* = 0.036) were associated with postinterventional delirium, whereas SbtO2, measured via NIRS, was not significant. Similarly, after the intervention, values of HbDeoxy (*p* = 0.042) and SbtO2 (*p* = 0.023) were significantly linked to the development of delirium, as shown in Figure 3.

## 4. Discussion

In this prospective observational study, we showed that CO and CBF increased after TAVR procedures. Despite the increase in CBF, we could not establish a connection between CBF changes and postinterventional delirium. In TAVR, the rate of patients developing delirium is still high, at 26–29%, although it is slightly lower than that in open cardiac surgery procedures [3,4]. While the technique has proven less invasive and more agreeable in critically ill patients, this does not seem to translate to delirium. One needs to consider the complex and multifactorial nature of postinterventional delirium [9]. Inflammation caused by cardiopulmonary bypass has long been suggested to be a factor associated with delirium. At the same time, bypass time could not be identified as a risk factor for delirium [10]. Excessive depth of anesthesia has also been shown to be associated with delirium [11]. Since neither cardiopulmonary bypass nor general anesthesia are present, the question of what leads to this relatively high rate of delirium in patients undergoing TAVR remains. Previously, we proposed an association between intraoperative hyperperfusion during cardiopulmonary bypass and postoperative delirium [5]. Cerebral hyperperfusion has mostly been described in the context of carotid artery surgery, in which it is a reperfusion phenomenon characterized by a breakthrough of blood flow through autoregulatory mechanisms after a sudden increase in flow due to the recanalization of the feeding vessel [12]. In the present study, we observed some degree of hyperperfusion, but this was not significantly associated with postinterventional delirium. At the same time, our exploratory analysis revealed an increased risk of delirium in patients with reduced cerebral oxygen saturation (SbtO2) and higher levels of deoxygenated hemoglobin (HbDeoxy) in the cerebral tissue after TAVR. Surprisingly, calculated cerebral oxygen delivery was not associated with delirium (Figure 3). These findings are not easy to interpret. It has been shown previously that the cerebral metabolic rate of oxygen can be uncoupled from CBF in certain disease states, thus providing a potential explanation for our results [13,14]. Although there were no differences in CBF between the delirium group and the non-delirium group, delirium was associated with reduced SbtO2 after TAVR, which may represent reduced cerebral metabolism, putting patients at risk of hyperperfusion. Therefore, a CBF increase after TAVR could still result in overperfusion in certain patients with low metabolic demands by overstressing the cerebral vasculature. However, post-TAVR HbDeoxy values were significantly decreased in patients with delirium. In a recent study, a relationship between cerebral desaturation during and after valve expansion and postinterventional delirium was shown, which is in line with our results [15]. However, the authors did not present a physiological rationale for their findings. Another explanation could be that the increase in CBF observed in our participants causes a reperfusion effect, stressing endothelial cells and causing secondary injury via local inflammation, which could be in line with a regional increase in oxygen consumption. Oxidative stress has been linked to delirium in cardiac surgery and hyperoxia and may play a role in TAVR [16]. Neuroinflammation due to reperfusion has been shown to be a complex cascade of endothelial and parenchymal cell activation, blood–brain barrier disruption, and expression of inflammatory mediators [17]. The development of delirium has been hypothesized to be linked to neuroinflammation [18]. Thus, an increase in CBF may cause reperfusion stress on previously chronically hypoperfused areas. In a recent study on patients undergoing cardiac surgery, we suggested a combination of reduced cerebral metabolism and hyperperfusion as a possible cause of postoperative delirium [19]. This neurovascular uncoupling may also apply in patients undergoing TAVR. Post-TAVR imaging could confirm this by identifying cerebral edema. Previous magnetic resonance imaging studies showed that patients with delirium after cardiac surgery or intensive care show signs of cerebral atrophy [20,21]. Another explanation lies in long-term adaptations of cerebral autoregulation to lower flow. One would expect a leftward shift of the autoregulation plateau in patients with aortic stenosis. With the rapid changes caused by TAVR, the upper limit of cerebral autoregulation may be exceeded. Time above the upper autoregulation limit has been shown to be associated with delirium in some patients undergoing cardiac surgery [22]. Although with our results, we are unable to support this view, such an effect cannot be ruled out with point measurements, as presented here. Preexisting cognitive impairment has been identified as one of the most prominent risk factors for delirium [9,10]. In our cohort, patients with lower MMSE scores had an increased risk of developing delirium. We also found that there was a significant, albeit weak, correlation between CBF and MMSE. Whether low MMSE is an actual risk factor or the result of chronic hypoperfusion remains unclear at this point, but it is in line with the mentioned findings of brain atrophy in delirium [20,21]. Other authors have hypothesized a connection between the microembolisms that may occur during TAVR and delirium. Cerebral microembolisms after TAVR were shown with magnetic resonance imaging [23]. A possible connection to the development of delirium was proposed by Nuebel et al., showing that neuron-specific enolase was increased in patients with delirium [24]. Our results neither contradict nor confirm these findings. While a connection between neuron-specific enolase and microembolisms appears plausible, other mechanisms may contribute to such results.

In our cohort of patients, we saw a CO-dependence of CBF, which was independent of MAP. Previously, van Houte et al. reported an increase in cardiac output after TAVR with unchanged carotid blood flow [2]. While we also observed an increase in CO, there was a concurrent increase in CBF, as indicated by ICG measurements. The reason for this disparity may be the different measurement methods. It has been shown that CO is an independent predictor of CBF. Skytioti et al. showed a positive relationship between CO and internal carotid blood flow in patients with cholecystectomy [25]. However, in Skytioti’s patients, internal carotid blood flow was also dependent on MAP. We also observed a positive relationship between CO and CBF, but the relationship between MAP and CBF was slightly negative. Thus, we can assume independence of the CBF from arterial driving pressure but dependence on the flow generated by CO after valve replacement. This may have implications for the evaluation of TAVR. A similar constellation—an increase in stroke volume and CO and a decrease in peripheral resistance—has been reported previously; these were interpreted as signs of successful valve replacement [26]. Hisdal et al. reported a relationship between CO, but not MAP, and precerebral blood flow in healthy patients during lower body negative pressure. Although one should consider the differences between healthy patients and our patients undergoing TAVR, it appears that CBF is exclusively dependent on CO. However, it is also possible that MAP was within the range of cerebral autoregulation, thus explaining the plateau in CBF, although it has to be mentioned that the plateau of Lassen’s cerebral autoregulation curve has recently been suggested to be more inclined than originally assumed [27,28]. A plausible explanation for the negative relationship between MAP and CBF in patients undergoing TAVR could also be a physiological mechanism to compensate for the increase in CO by reducing peripheral resistance, possibly mediated by the baroreflex. However, this remains to be confirmed by studies.

There are several limitations associated with this study. It is possible that a certain number of patients with delirium were not identified in our cohort, especially during nighttime and in cases of hypoactive delirium, which is much harder to detect. This is a general weakness of tests for delirium that has been suggested previously and cannot be addressed easily [24]. Another factor to consider is that delirium is known to be of multifactorial origin, thus making the interpretation of relationships difficult [29]. Finally, CBF measurements represent singular measurements at two time points and may not represent the complete dynamics of cerebral blood flow after TAVR. This is due to the nature of CBF measurement, which requires an ICG concentration curve. Therefore, CBF values represent static rather than dynamic parameters.

In conclusion, in our cohort of patients undergoing TAVR, we found a concurrent increase in CBF and CO, with a slight decrease in MAP. Our results suggest that CO is the determining factor for CBF in these patients. Our results do not suggest a relationship between CBF changes and delirium after TAVR but may still be in line with a cerebral perfusion mismatch in these patients.

## Figures and Tables

**Table 1:** Patient demographics for all patients and patients with and without delirium. Values are means ± standard deviation unless stated otherwise. MMSE: Mini-Mental State Examination, SD: standard deviation.

Parameter	All Patients (n = 52)	No Delirium (n = 39)	Delirium (n = 13)
Age [y, ± SD]	80.6 (±7.2)	79.8 (±7.6)	82.8 (±5.8)
Gender			
female [n, %]	22 (42.3%)	18 (46.2%)	4 (30.8%)
male [n, %]	30 (57.7%)	21 (53.8%)	9 (69.2%)
weight [kg, ±SD]	76.6 (±16.6)	76.8 (±17.4)	76.1 (±13.3)
height [cm, ±SD]	169.0 (±10.2)	168.7 (±10.8)	170.5 (±8.9)
MMSE [±SD]	26.9 (±2.9)	27.5 (±2.4)	25.2 (±2.5)

**Table 2:** CBF and CBV values before and after TAVR in the non-delirium and delirium groups. CBF: cerebral blood flow, CBV: cerebral blood volume, CO: cardiac output, MAP: mean arterial pressure.

	No Delirium (n = 39)			Delirium (n = 13)		
	Pre-TAVR	Post-TAVR	∆ (%)	Pre-TAVR	Post-TAVR	∆ (%)
CBV (mL/100 g)	4.3 (±1.8)	4.7 (±2.9)	9	4.8 (±2.5)	5.1 (±3.7)	6
CBF (mL/100 g/min)	18 (±11)	26 (±20)	44	16 (±13)	23 (±16)	44
CO (L/min)	4.5 (±1.2)	5.5 (±1.6)	22	4.7 (±1.2)	5.5 (±2.2)	17
MAP (L/min)	93 (±12)	85 (±12)	8	95 (±17)	84 (±14)	12

## Data Availability

Raw data are provided in the Supplement S1.

## Supplementary Materials

The following supporting information can be downloaded at: https://www.mdpi.com/article/10.3390/jcm14124317/s1, Supplemental Tables S1–S3: linear models, Supplement S1: raw data.
