# Supplementary material for: Regional Cerebral Blood Flow Increase After Transcatheter Aortic Valve Replacement Is Related to Cardiac Output but Is Not Associated with Delirium: An Observational Cohort Study Using Transcranial Indocyanine Green Dye Dilution Technique
Source: J Clin Med. 2025 Jun 17;14(12):4317. doi: 10.3390/jcm14124317 (PMC12194422; doi:10.3390/jcm14124317)
Supplement: Supplementary file 1 [file jcm-14-04317-s001.zip › Tables S1-S3.pdf]

Supplemental Table S1: Association between cerebral blood flow (CBF) at baseline (independent variable) and the mini-mental-status-examination (MMSE , outcome) analyzed by a linear regression model.

	**Outcome variable: MMSE**		
Predictors	*Estimates*	*CI*	*p*
(Intercept)	25.12	23.46 – 26.77	**<0.001**
CBF at baseline [ml/100g/min]	0.10	0.02 – 0.18	**0.015**
Observations	43		
R^2 / R^2 adjusted	0.135 / 0.114		

Supplemental Table S2: Association between cardiac output (CO, independent variable) and cerebral blood flow (CBF, outcome) analyzed by a linear mixed model.

	**Outcome variable: CBF**		
Predictors	*Estimates*	*CI*	*p*
(Intercept)	9.67	-1.81 – 21.16	0.098
CO [l/min]	2.33	0.15 – 4.52	**0.037**
**Random Effects**			
σ^2	161.26		
τ_{00}	83.22 $_{id}$		
ICC	0.34		
N	49 $_{id}$		
Observations	87		
Marginal R^2 / Conditional R^2	0.054 / 0.376		

Supplemental Table S3: Association between mean arterial pressure (MAP, independent variable) and cerebral blood flow (CBF, outcome) analyzed by a linear mixed model.

	**Outcome variable: CBF**		
Predictors	*Estimates*	*CI*	*p*
(Intercept)	26.57	1.07 – 52.07	**0.041**

MAP [mmHg]	-0.06	-0.34 – 0.22	0.672
**Random Effects**			
σ^2	178.87		
τ_{00}	77.20 $_{id}$		
ICC	0.30		
N	47 $_{id}$		
Observations	86		
Marginal R^2 / Conditional R^2	0.002 / 0.303		
